# Stim2-Eb3 Association and Morphology of Dendritic Spines in Hippocampal Neurons

**DOI:** 10.1038/s41598-017-17762-8

**Published:** 2017-12-15

**Authors:** Ekaterina Pchitskaya, Nina Kraskovskaya, Daria Chernyuk, Elena Popugaeva, Hua Zhang, Olga Vlasova, Ilya Bezprozvanny

**Affiliations:** 1 Laboratory of Molecular Neurodegeneration, Department of Medical Physics, Peter the Great St. Petersburg Polytechnic University, St. Petersburg, Russian Federation; 2 Department of Physiology, UT Southwestern Medical Center at Dallas, Dallas, TX 75390 USA

## Abstract

Mushroom spines form strong synaptic contacts and are essential for memory storage. We have previously demonstrated that neuronal store-operated calcium entry (nSOC) in hippocampal neurons is regulated by STIM2 protein. This pathway plays a key role in stability of mushroom spines and is compromised in different mice models of Alzheimer’s disease (AD). Actin was thought to be the sole cytoskeleton compartment presented in dendritic spines, however, recent studies demonstrated that dynamic microtubules with EB3 capped plus-ends transiently enter spines. We showed that STIM2 forms an endoplasmic reticulum (ER) Ca^2+^ -dependent complex with EB3 via Ser-x-Ile-Pro aminoacid motif and that disruption of STIM2-EB3 interaction resulted in loss of mushroom spines in hippocampal neurons. Overexpression of EB3 causes increase of mushroom spines fraction and is able to restore their deficiency in hippocampal neurons obtained from PS1-M146V-KI AD mouse model. STIM2 overexpression failed to restore mushroom dendritic spines after EB3 knockdown, while in contrast EB3 overexpression rescued loss of mushroom spines resulting from STIM2 depletion. We propose that EB3 is involved in regulation of dendritic spines morphology, in part due to its association with STIM2, and that modulation of EB3 expression is a potential way to overcome synaptic loss during AD.

## Introduction

Synapse is an extremely important morphological part of neuron formed by presynaptic axon ending and by postsynaptic dendritic spine. There is a lot of evidence that alterations in synapse morphology and density in different brain regions accompany many types of neurodegenerative and psychiatric disorders^1^ such as Alzheimer’s Disease (AD)^2,3^, Huntington’s Disease^4,5^, Parkinson’s disease^6^, autism^7^ and depression^8^. Dendritic spines are divided into three big groups according to their morphology: mushroom spines, stubby spines and thin spines^9^. It is believed that mushroom spines, which have large “head” and express big number of neurotransmitter receptors and ion channels on their surface, form functionally stronger synapses and act as sites of memory storage^10^. Elimination of mushroom spines has been proposed to underlie the memory loss observed in AD patients^11–13^. Indeed, in our latest studies, we demonstrated the reduction of mushroom spines fraction in PS1-M146V-KI^14^, APP-KI^15,16^ mice models of AD and in conditions of low amyloid toxicity^17^. What is the reason for spine loss? Recently we have showed that proper functioning of neuronal store-operated calcium entry (nSOC) in postsynaptic spines is necessary for stability of mushroom dendritic spines in hippocampal neurons, and that downregulation of this pathway leads to mushroom spine loss observed in diverse AD models.

Store-operated calcium entry is controlled by Stromal interacting molecules (STIMs) – an endoplasmic reticulum (ER) calcium sensor proteins, containing amino-terminal EF-hand Ca^2+^ -binding domain located in the ER lumen^18^. STIM family includes STIM1 and STIM2 proteins. STIM2 binds Ca^2+^ with lower affinity than STIM1 and it plays a role of homeostatic Ca^2+^ modulator^19^. When ER Ca^2+^ concentration drops, STIMs oligomerize and travel to ER-plasma membrane (PM) junctions, where they bind calcium-conducting channels from Orai and/or TRPC families and trigger Ca^2+^ entry into cytoplasm^20–22^. An existence of ER-PM contact sites in the spines has been recently confirmed by application of focused ion beam-scanning electron microscopy^23^. nSOC is now considered as not only store refilling mechanism but also as an vital signaling pathway participating in the important neuronal processes^24^. In previous studies role of STIM1 and STIM2 in supporting nSOC was demonstarted^25–27^. Functional studies of nSOC in hippocampal neurons suggested a critical role of Orai1 channels^28^. Activation of STIM1 and nSOC was described in hippocampal neurons following activation of type I metabotropic glutamate receptors (mGluR) or muscarinic acetylcholine receptors (mAChR)^29^. Notably, nSOC-independent functions of STIM1 in hipocampal neurons were also reported. It was suggested that STIM1 directly controls levels of phosphorylation and surface expression of the AMPAR^30^. The role of STIM1 in control of neuronal L-type Ca^2+^ channel activity^31^ and feedback regulation of Ca^2+^ signals in presynaptic terminals was reported^32^. Consistent with important role played by STIM1 and STIM2 in neurons, learning and memory phenotypes were observed following knockout or overexpression of these proteins^30,33,34^. In addition, it was shown that neurons from STIM2 knockout markedly protected from neurological damage in a model of focal cerebral ischemia^35^. Recent studies suggested that nSOC plays an important role in the context of neurodegeneration. Downregulation of STIM2 proteins was observed in Alzheimer’s disease samples^14,36^. nSOC impairments were reported for Alzheimer’s^14,15^, Huntington’s^37^ and Parkinson’s^38,39^ diseases. In case of AD, overexpression of nSOC component STIM2 prevents mushroom spine loss in AD mouse models and in conditions of amyloid toxicity suggesting that downregulation of STIM2-nSOC pathway is a potential mechanism of synaptic loss in AD^14,15,17^.

It is generally known that dynamic structure of dendritic spines is maintained by actin filaments^9,23^, while microtubules (MTs) are cytoskeleton-organizing components in more stable parts of neuron such as axons and dendritic branches^24,40^. Nevertheless, the recent studies revealed that dynamic microtubules may enter dendritic spines in the activity-dependent manner^37,41–44^ and these entries trigger the enlargement of spines head^43^. The frequency of MTs entry into spines and the number of targeted spines increases after the induction of long-term potentiation (LTP)^43^. Thus, invasions of MTs into spines appear to be involved in the synaptic plasticity mechanisms. Dynamic microtubule plus-end is decorated by so-called end-binding proteins (EB), which are presented by three homologues EB1, EB2 and EB3^45^. It was shown that EB1 forms complex with STIM1 protein and mediates ER movement in non-excitable cells^46,47^. EB3 protein is expressed abundantly in the nervous system and enters the dendritic spines at the tip of growing microtubule^37,41–44^. In the present study, we set out to determine if STIM2, a neuronal-specific homologue of STIM1, interacts with EB3 protein in hippocampal neurons, and what effect this interaction exerts on dendritic spines morphology. The results obtained in our experiments demonstrate that EB3 interacts with STIM2 and that this interaction promotes formation of mushroom spines in hippocampal neurons. Moreover, EB3 overexpression prevented loss of mushroom spines in PS1-M146V-KI AD mice model neurons. Therefore, our findings open a new potential way of stabilizing synaptic spines in AD.

## Results

### STIM2 interacts with EB3 in synapses through SxIP motif in a ER Ca^2+^ concentration-dependent manner

**Figure 1:**
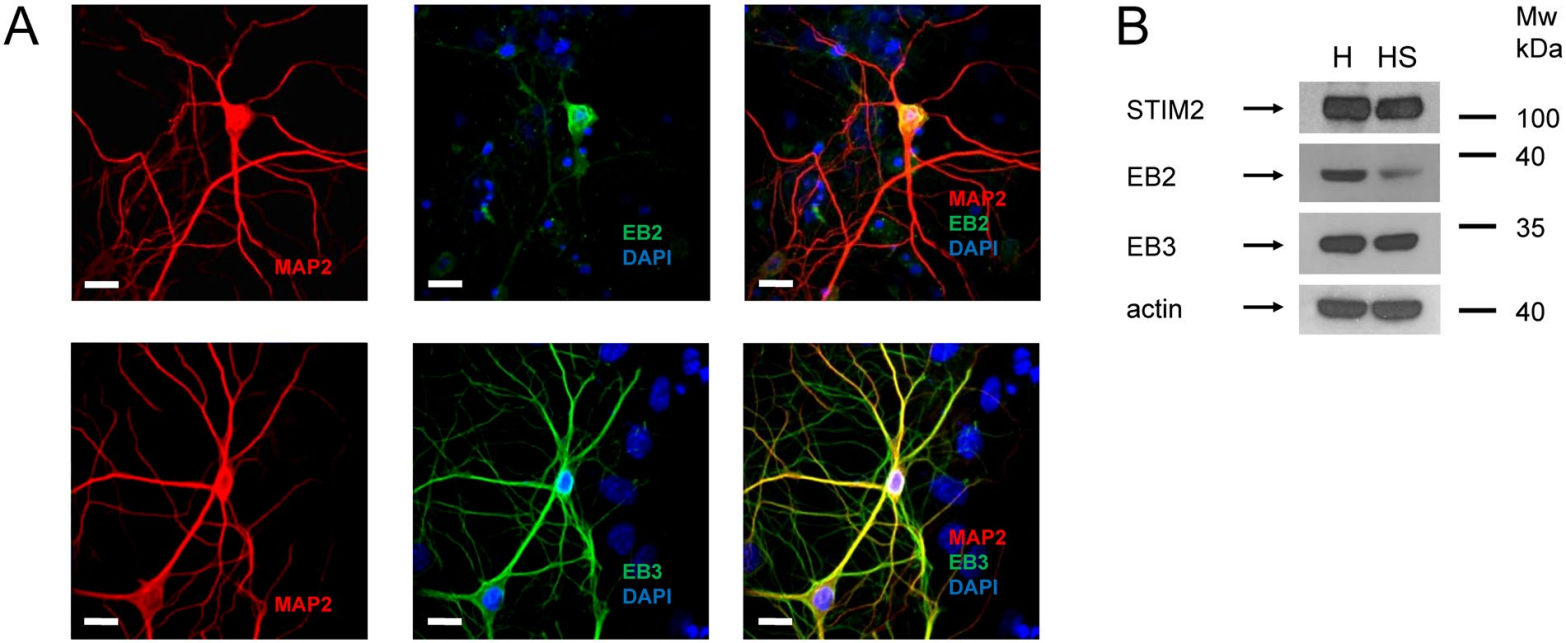
EB2/3 distribution in neuron. (**A**) Subcellular localization of EB3 and EB2 were analyzed by immunostaining of WT hippocampal primary cultures with anti-EB2/3 antibody (green). MAP2 (red) was used for neuronal labeling and DAPI (blue) was used for identification of cell nucleus. Scale bar corresponds to 10 μm. (**B**) Western blot analysis of STIM2, EB2, EB3 proteins expression levels in hippocampal (H) and crude hippocampal synaptosome fraction (HS) lysates from 3-month-old wild-type mice. Actin was used as loading control. Representative results from three independent experiments are shown. Full-length blots are presented in Supplementary Figure S1.

There are three isoforms of EB proteins in mammals – EB1, EB2 and EB3. It has been reported that they all are expressed in the nervous systems, with EB2 and EB3 enriched in hippocampal region (Fig. S4). To understand roles of EB2 and EB3 in hippocampal neurons, we performed series of immunostaining experiments with primary hippocampal neuronal cultures. In these experiments, we discovered that EB2 protein is highly enriched in soma in comparison to dendrites, whereas EB3 is broadly expressed in both soma and dendrites (Fig. 1A). Using a different approach, we performed comparison of EB2, EB3 and STIM2 proteins expression in whole hippocampus and in crude hippocampal synaptosome fraction lysates using Western blot. In these experiments we discovered that EB3 and STIM2 are present at synaptic locations, while EB2 was significantly reduced in synaptosomal fraction (Fig. 1B). Dendritic and synaptic localization of EB3 in hippocampal neurons is consistent with the previous reports^37,41–44^. Synaptic localization of STIM2 is in agreement with our previous findings^14^.

**Figure 2:**
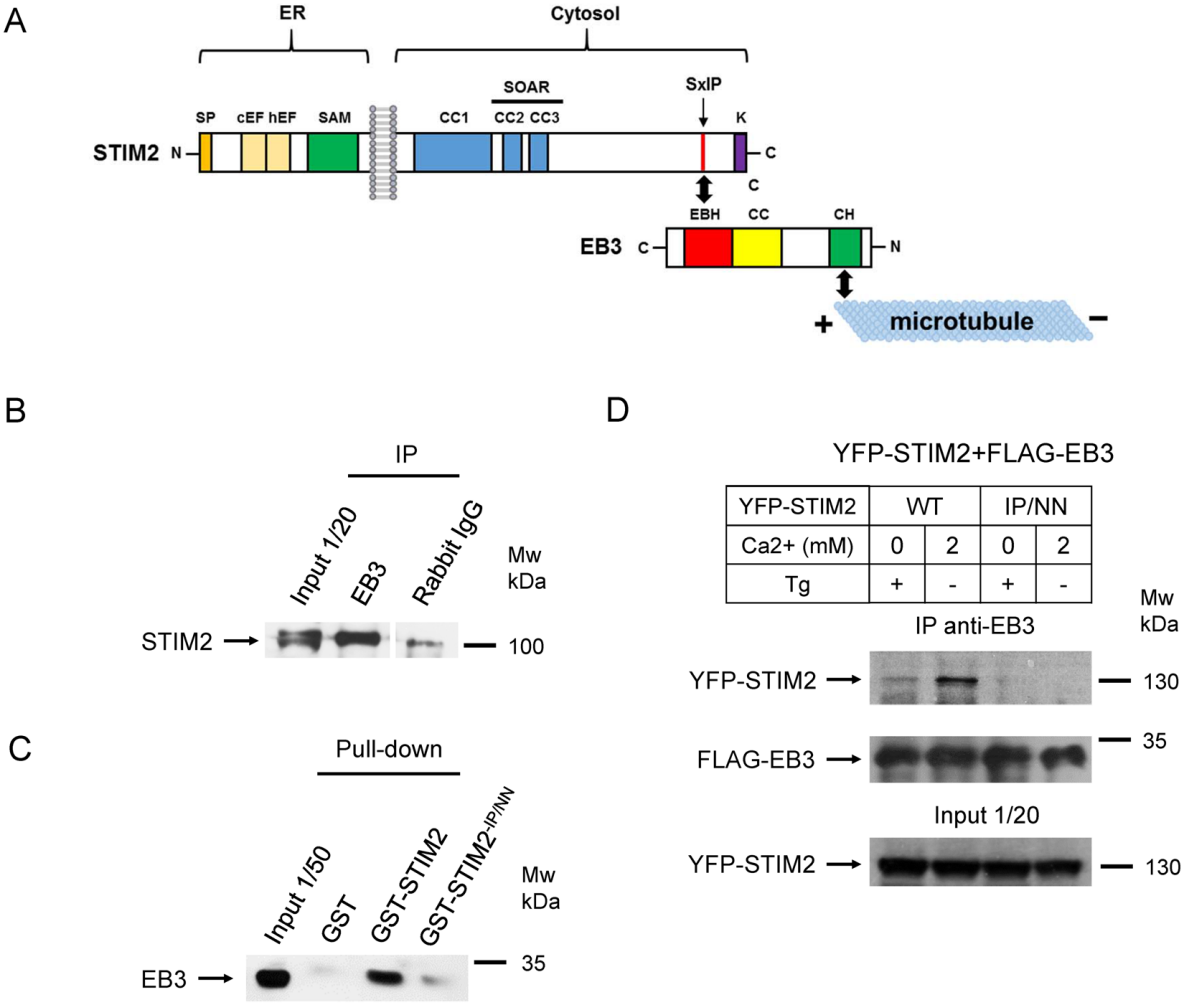
STIM2 interacts with EB3 via SGIP aminoacid domain. (**A**) A scheme of STIM2 and EB3 protein structure with indication of possible interaction site. Abbreviations: SP – signal peptide; c/hEF – canonical and hidden EF hand; SAM –sterile alpha motif; CC – coiled coil domain; SOAR – STIM-Orai activating region;SxIP – Ser-x-Ile-Pro tetrapeptide motif; K –lysine-rich domain, EBH – EB homology domain, CH – calponin homology domain. (**B**) Immunoprecipitation from whole brain crude synaptosomal fraction lysates with rabbit polyclonal antibody against EB3 or a control rabbit serum. Input is 1/10 of lysate used for immunoprecipitation. (**C**) Pull-down analyses of interactions between GST, GST-STIM2-CT and GST-STIM2-IP/NN recombinant proteins and crude whole brain synaptosomal fraction lysates from 3-month-old WT mice. Input is 1/50 of lysate used for pull-down experiments. (**B**,**C**) Representative results from three independent experiments are shown. Cropped blots are displayed, full-length blots are presented in Supplementary Figure S1. (**D**) Immunoprecipitation from HEK293T cells lysates with rabbit polyclonal antibody against EB3 or a control rabbit serum. HEK293T cell were transfected with FLAG-EB3 and YFP-STIM2 or YFP-STIM2-IP/NN. Cells were preincubated in Ca^2+^-free aCSF with addition of 1 μM Thapsigargin (+Tg) or in aCSF containing 2 mM Ca^2+^ (-Tg) for 10 minutes and lysed. Level of YFP-STIM2 bound to FLAG-EB3 was evaluated by immunoblotting using an anti-STIM2 antibody. The level of pulled-down FLAG-EB3 was evaluated with an anti-FLAG antibody. Input is 1/10 of lysate used for immunoprecipitation.

Taking into account that both STIM2 and EB3 proteins are present at synaptic locations, we focused on their possible interaction. Co-immunoprecipitation from synaptosomal lysates confirmed the association of EB3 and STIM2 proteins in physiological conditions (Fig. 2B). STIM2 is a multidomain transmembrane protein, with N-terminus located in the ER lumen and the C-terminus in the cytoplasm (Fig. 2A). STIM1 is a homologue of STIM2 that shares the same domain structure^48^. It has been established that STIM1 and EB1 proteins bind to each other via interaction between S/TxIP aminoacid motif in C-terminus of STIM1 and EB-homology domain of EB1 (Fig. 2A)^46,47^. A double mutation in STIM1 S/TxIP motif replacing Ile-Pro dipeptide by Asn-Asn (I644N, P645N) disrupted association between STIM1 and EB1^47^. Analysis of STIM2 sequence revealed that it also contains SxIP motif (686–689 aminoacid residues) (Fig. 2A). To confirm that STIM2-EB3 association is mediated by SxIP motif, we performed pull-down experiments with glutathione S-transferase (GST)-fusion proteins of the wild type STIM2 cytoplasmic part (GST-STIM2-CT) and GST-STIM2-IP/NN-CT protein, that contains I688N and P689N mutations within SxIP motif of STIM2 (Fig. S3). Corresponding GST-fusion proteins were incubated with synaptosomal lysates and the isolated proteins were analyzed by Western blot with anti-EB3 antibody. We discovered that STIM2-CT domain strongly associates with EB3 protein, and this association is disrupted by IP/NN mutation (Fig. 2C).

To further extend our findings, we investigated if EB3 and STIM2 interaction depends on ER Ca^2+^ levels as has been reported previously for EB1 and STIM1 association^49^. In order to test this idea, we performed immunoprecipitation experiments with anti-EB3 antibodies using lysates from HEK293T cells transfected with FLAG-EB3 and YFP-STIM2 plasmids. Сells were incubated in Ca^2+^-free aCSF with addition of 400 µM EGTA and 1 µM Thapsigargin for 10 minutes before lysis to achieve ER calcium store depletion. Control cells were incubated in normal aCSF containing 2 mM Ca^2+^ for the same duration. We found that YFP-STIM2 forms complex with FLAG-EB3 in HEK293T cells (Fig. 2D). Consistent with GST pull-down experiments, YFP-STIM2-IP/NN mutant did not associate with FLAG-EB3 (Fig. 2D). ER calcium store depletion disrupted association of YFP-STIM2 and FLAG-EB3 proteins (Fig. 2D), similar to the effects observed for STIM1 and EB1 isoforms^49^. From these experiments we concluded that STIM2 interacts with EB3 through SxIP motif in ER Ca^2+^-dependent manner.

### Disruption of STIM2-EB3 interaction causes loss of hippocampal mushroom spines

**Figure 3:**
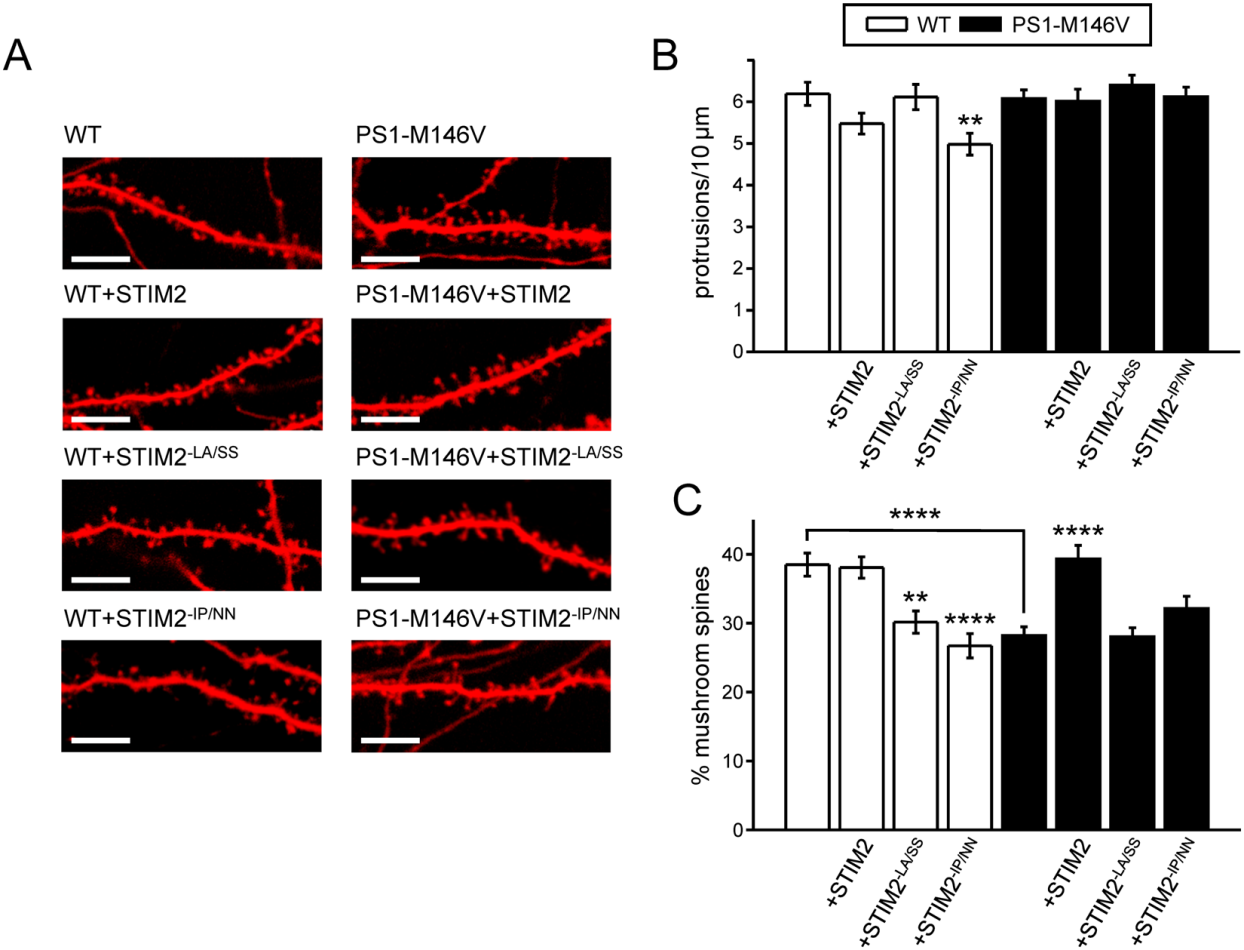
Disruption of EB3-STIM2 interaction leads to elimination of mushroom dendritic spines in primary hippocampal cultures. (**A**) Confocal images of WT and PS1-M146V-KI hippocampal neurons transfected with tdTomato or cotransfected with tdTomato and STIM2, STIM2-LA/SS, STIM2-IP/NN plasmids at DIV7 and fixed at DIV17. Scale bar corresponds to 10 μm. (**B**,**C**) The number of protrusions per 10 μm dendrite length (**B**) and average fraction of mushroom spines (**C**) for each group of cells shown on panel A are presented as mean ± SEM (n ≥ 20 neurons from 3 batches of cultures). **p < 0.01; ****p < 0.0001.

We previously shown that overexpression of STIM2 can rescue mushroom spine defects in primary hippocampal neurons from PS1-M146V-KI mouse AD model^14^. Further we demonstrated that STIM2 protein with L377S and A380S mutations in SOAR (the STIM1 Orai activating region) domain (STIM2-LA/SS), which disrupt association of STIM2 with Orai channels, failed to rescue mushroom spines pathology observed in PS1-M146V-KI neurons^50^. This finding indicates that mutant STIM2-LA/SS is not functional. We utilized the similar approach to evaluate effects of IP to NN mutations STIM2. To test how STIM2-IP/NN mutant will influence dendritic spines morphology in comparison to wild type STIM2, we co-transfected primary hippocampal cultures from WT and PS1-M146V-KI mice with tdTomato plasmid and plasmids coding STIM2, STIM2-LA/SS and STIM2-IP/NN at DIV7. The neurons were fixed at DIV17 and imaged by confocal microscopy. Obtained confocal images were used to quantify the density of dendritic protrusions and the percent of mushroom spines by using an automated scoring procedure (see Methods section for details). Consistent with the previous findings^14^, we observed a significant reduction in the fraction of mushroom spines in PS1-M146V-KI cultures when compared with WT (Fig. 3A). On average, the fraction of mushroom spines was equal to 38.5 ± 1.7% (n = 20) for WT cultures and to 28.2 ± 1.1% (n = 20, *P* = 0.00006) for PS1-M146V-KI cultures (Fig. 3C). Consistent with previous results^14^, total spine density was not affected by PS1-M146V mutation (Fig. 3B). Overexpression of STIM2 resulted in rescue of mushroom spines fraction in PS1-M146V-KI cultures to 39.3 ± 1.9% (n = 18, p = 0.00007) (Fig. 3A,C). In agreement with previous findings^50^, expression of STIM2-LA/SS mutant failed to rescue mushroom spine deficiency in PS1-M146V-KI cultures and reduced the fraction of mushroom spines in WT cultures to 30.1 ± 1.6% (n = 18, p = 0,00005) (Fig. 3A,C). Similarly, overexpression of STIM2-IP/NN mutant induced the reduction of mushroom spines fraction in WT neurons to 26.7 ± 1.8% (n = 21, P = 0.00005) and was not able to restore reduced percent of mushroom spines in PS1-M146V-KI neurons (Fig. 3A,C). Moreover, STIM2-IP/NN overexpression in WT cultures resulted in decrease in protrusions density from 6.2 ± 2.8 (n = 20) protrusions per 10 µm dendrite length to 5 ± 2.63 protrusions per 10 µm (n = 21, P = 0.003, Fig. 3B). These results support the important role of STIM2 and EB3 interaction in regulation of hippocampal mushroom spines morphology.

### EB3 and STIM2 are necessary for normal dendritic spines morphology

**Figure 4:**
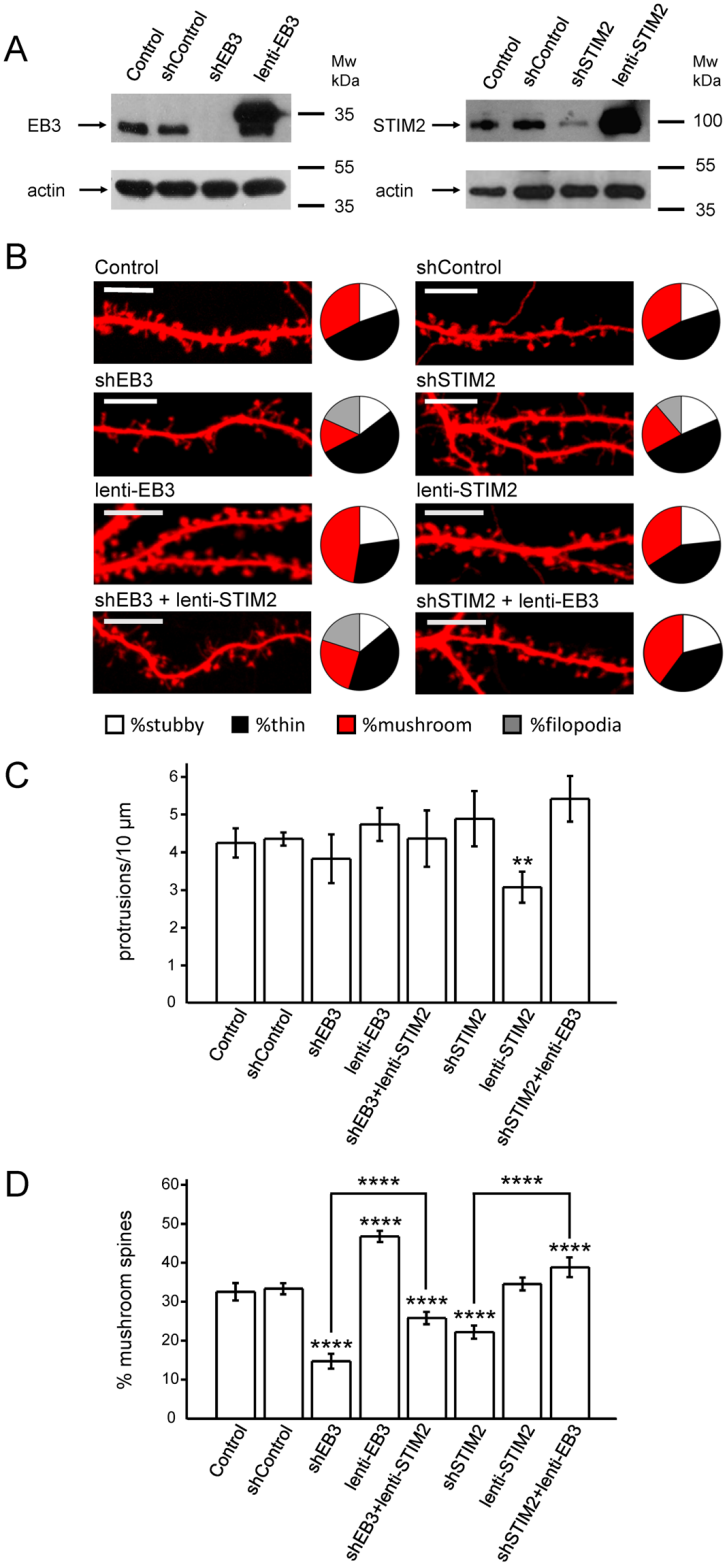
EB3 and STIM2 are necessary for normal mushroom dendritic spines morphology. (**A**) Western blot analysis of STIM2 and EB3 proteins expression levels in lysates from WT hippocampal primary cultures (Control) infected with lentiviruses encoding control RNAi (shControl), RNAi against STIM2 (shSTIM2), STIM2 (lenti-STIM2), RNAi against EB3 (shEB3), EB3 (lenti-EB3). Actin was used as loading control. (**B**) Confocal images of WT hippocampal neurons transfected with tdTomato (Control) at DIV7 and fixed at DIV17 and infected with lentiviruses encoding control RNAi (shControl), RNAi against STIM2 (shSTIM2), RNAi against EB3 (shEB3), STIM2 (lenti-STIM2), EB3 (lenti-EB3) or both shSTIM2 + lenti-EB3, shEB3 + lenti-STIM2. Pie charts illustrate distribution of dendritic spines between morphological groups, where black color matches mushroom spines, red – thin spines, white – stubby spines, grey – filopodia protrusions. Scale bar corresponds to 10 μm. (**C**,**D**) The number of protrusions per 10 μm dendrite length (**C**) and average fraction of mushroom spines (**D**) for each group of cells shown on panel (B) are presented as mean ± SEM (n ≥ 20 neurons from 3 batches of cultures). **p < 0.01; ****p < 0.0001.

To future investigate the function of EB3 and STIM2 in hippocampal spines, we performed overexpression and knockdown of these proteins in hippocampal cultures with help of lentivirus coding full protein sequence or targeting short hairpin RNA (shRNA). Lentiviruses encoding off target shRNA were used in control experiments (shControl). Efficiency of all assembled lentiviruses was validated by Western blot (Fig. 4A). Wild type hippocampal cultures we transfected with tdTomato plasmid at DIV7 and infected on DIV8 with lentiviruses encoding EB3, STIM2, shEB3, shSTIM2 and shControl. Cells were fixed at DIV17 and dendritic spine morphology for each experimental group was analyzed with help of confocal imaging (Fig. 4B). Automated analysis of spines shape revealed that the fraction of mushroom spines was significantly reduced following shRNA-mediated knockdown of EB3 protein, with proportional increase of thin spines and even filopodium-like protrusions (Fig. 4B,D). In mature DIV17 wild type control neurons filopodias were absent. Consistent with the previous findings^14^, knockdown of STIM2 protein also resulted in reduction of mushroom spines fraction, although effects were less dramatic than with shEB3 knockdown (Fig. 4B,D). On average, the fraction of mushroom spines was equal 32.5 ± 2.3% (n = 21) for WT neurons, 33.3 ± 1.4% (n = 21) for WT neurons infected with control shRNA, 14.7 ± 1.9% (n = 21) for shEB3 infected neurons (P < 0.0001) and 22.2 ± 1.7% (n = 21) for shSTIM2 infected neurons (P < 0.0001) (Fig. 4D). In contrast, overexpression of EB3 protein induced an increase in the fraction of mushroom spines to 46.5 ± 1.4% (n = 21, P < 0.0001), so they became a dominant fraction of dendritic spines (Fig. 4B,D). Dendritic spine effects of EB3 knockdown and overexpression are consistent with the previous reports^37,51^. STIM2 overexpression did not significantly change the fraction of mushroom spines in hippocampal neurons yielding 34.3 ± 1.6% (Fig. 4B,D). STIM2 overexpression resulted in decreased protrusions density from 4.42 ± 0.15 protrusions per 10 µm for control group (n = 21) to 3.03 ± 0.16 protrusions per 10 µm (n = 21, P = 0.006, Fig. 4C). Interestingly, EB3 protein overexpression was sufficient to rescue mushroom spine fraction following STIM2 knockdown (Fig. 4B). On average, mushroom spines percent in neurons co-infected with Lenti-shSTIM2 and Lenti-EB3 viruses was equal to 38.8 ± 2.5% (n = 21, P < 0.0001) (Fig. 4D). In contrast, overexpression of STIM2 protein resulted only in partial rescue of mushroom spines following knockdown of EB3 protein (Fig. 4B). Particularly, mushroom spines fraction in neurons co-infected with Lenti-shEB3 and Lenti-STIM2 viruses was equal to 25.8 ± 1.6% (n = 21, P < 0.0001) (Fig. 4D).

### Modulation of dendritic spine nSOC by EB3 protein

**Figure 5:**
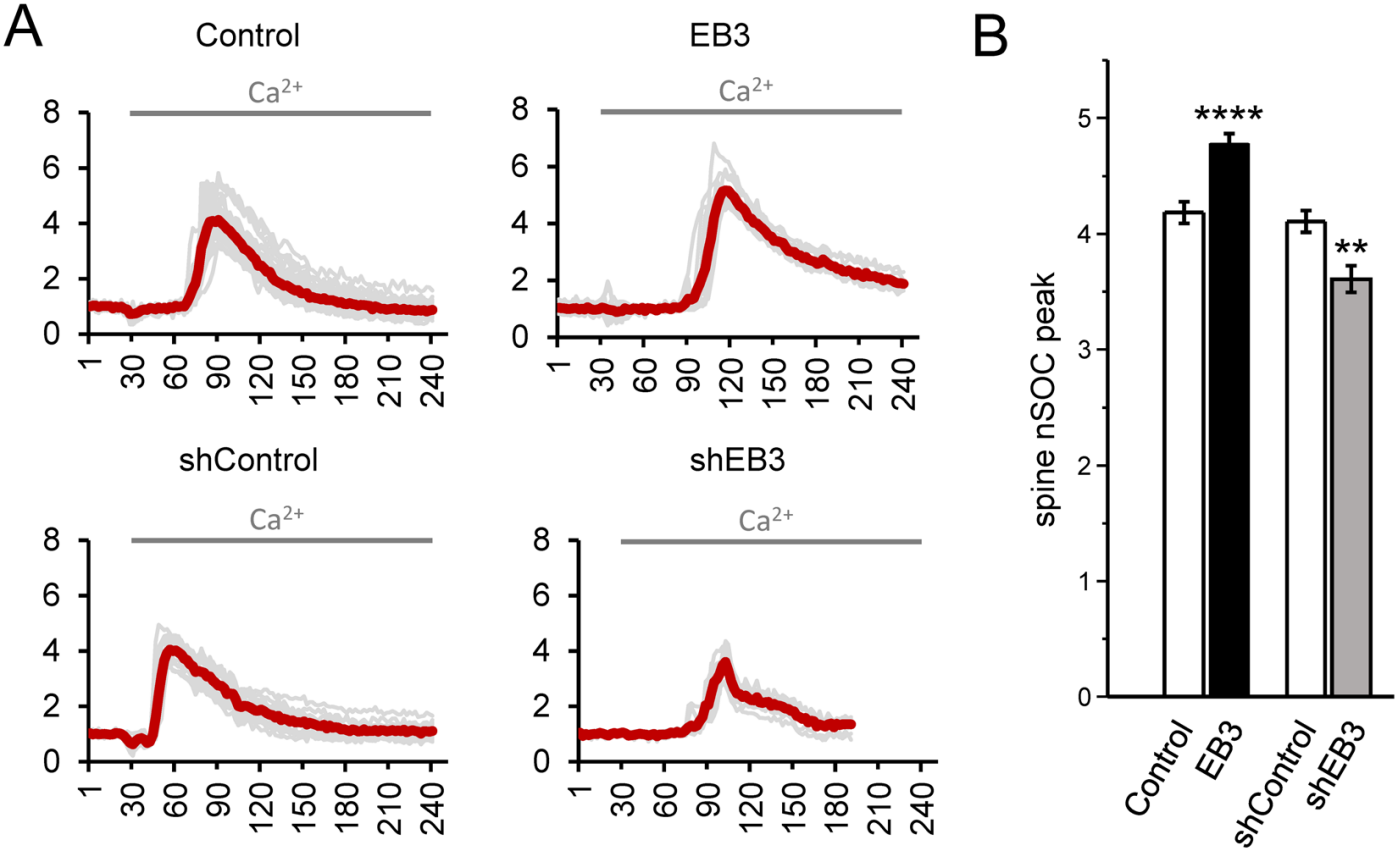
EB3 modulates dendritic spines nSOC in primary hippocampal cultures. (**A**) Synaptic GCaMP5.3 Ca^2+^ signals (F/F_0_) are shown for DIV15 WT hippocampal neurons transfected with GCaMP5.3 or co-transfected with GCaMP5.3 and FLAG-EB3, shControl, shEB3-EB3 plasmids at DIV7. (**B**) Average synaptic nSOC peak for each group of cells shown on panel A is presented as mean ± SEM (n ≥ 117 spines from 3 to 6 batches of cultures). **p < 0.01; ****p < 0.0001.

In our recent studies, we discovered that nSOC plays a key role in supporting stability of mushroom dendritic spines in hippocampal neurons^14,15^. In this study, we demonstrated that manipulating EB3 protein expression levels significantly affects percent of mushroom dendritic spines, and therefore rises a question if it influences dendritic spine nSOC. To answer this question we performed Ca^2+^ imaging experiments in dendritic spines using genetically encoded calcium sensor GCaMP5.3. In these experiments, WT hippocampal neurons were transfected with GCaMP5.3 expression plasmid or co-transfected with GCaMP5.3 and shControl, shEB3 and FLAG-EB3 plasmids at DIV7, and Ca^2+^ imaging experiments were performed at DIV15. GCaMP5.3 fluorescence signal was used to visualize spine structures and to image changes in spine Ca^2+^ concentration, as described previously in our studies with PS1-M146V-KI^14^ and APP-KI neurons^15^. In order to deplete ER Ca^2+^ stores in the dendritic spines, transfected neurons were incubated in Ca^2+^ free aCSF for 30 minutes in the presence of 1 µM Thapsigargin and 0.4 mM EGTA and with addition of calcium channels inhibitor cocktail (see Methods section for details). After baseline recording nSOC was evoked by puff application of 5 µl 2 M Ca^2+^ in Ca^2+^ free aCSF and spine signals were recorded as changes in GCaMP5.3 fluorescence (Fig. 5A). In agreement with our previous findings, we observed robust spine nSOC signal in WT neurons^14,15^ (4.2 ± 0.1, n = 121, Fig. 5B). EB3 overexpression induced slight but significant increase in nSOC spines signals to 4.8 ± 0.1 (n = 230, P < 0.0001, Fig. 5B), while EB3 depletion reduced spine nSOC level to 3.6 ± 0.1 (n = 117, P = 0.001, Fig. 5B).

### EB3 overexpression rescues mushroom spine loss in PS1-M146V-KI primary hippocampal neurons

**Figure 6:**
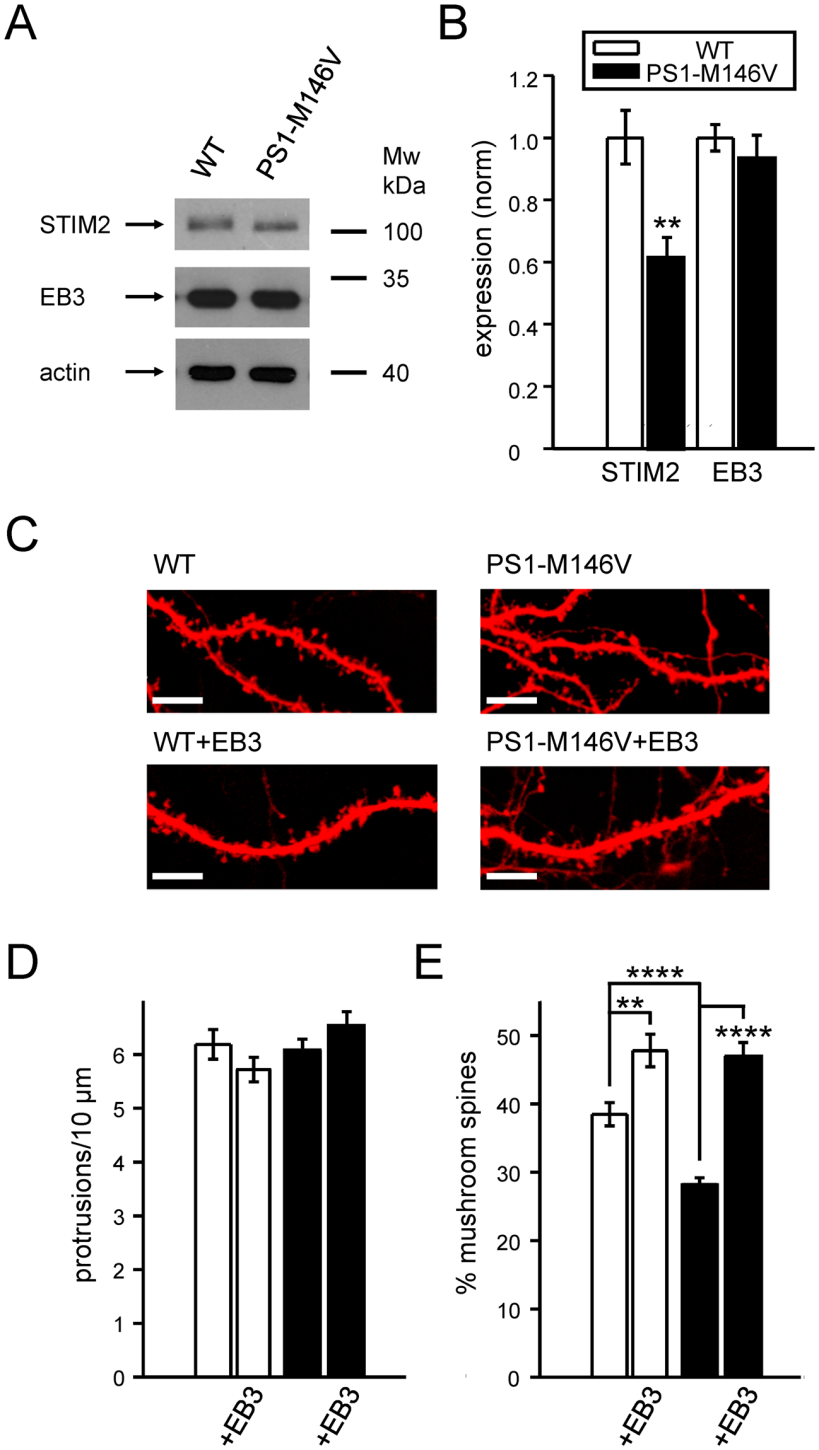
EB3 overexpression rescues mushroom spines in PS1-M146V-KI primary hippocampal cultures. (**A**) Western blot analysis of STIM2 and EB3 proteins expression levels in lysates from WT and PS1-M146V-KI hippocampal primary cultures. Actin was used as loading control. Representative results from 3 independent cultures are shown. Cropped blots are displayed, full-length blots are presented in Supplementary Figure S1. (**B**) Quantification of STIM2 and EB3 expression levels in WT and PS1-M146V-KI hippocampal primary cultures. The mean density of each band was normalized to actin signal in the same sample and averaged. These values were normalized to WT for every batch of cells. Values are shown as mean ± SEM (n = 3 batches of culture). **p < 0.01. (**C**) Confocal images of WT and PS1-M146V-KI hippocampal neurons transfected with tdTomato or cotransfected with tdTomato and FLAG-EB3 at DIV7 and fixed at DIV17. Scale bar corresponds to 10 μm. (**D**,**E**) The number of protrusions per 10 μm dendrite length (**D**) and average fraction of mushroom spines (**E**) for each group of cells shown on panel (**C**) are presented as mean ± SEM (n ≥ 21 neurons from 3 batches of cultures). **p < 0.01; ****p < 0.0001.

In previous studies, we discovered that the alterations in spine nSOC and subsequent loss of mushroom spines in PS1-M146V-KI neurons are due to reduction in expression levels of STIM2 protein^14^. Is expression of EB3 protein also affected in PS1-M146V-KI neurons? To answer this question, we performed a series of Western blot experiments with lysates prepared from DIV17 WT and PS1-M146V-KI primary hippocampal cultures. Consistent with the previous findings^14^, we observed 38% reduction of STIM2 levels in PS1-M146V-KI cultures (n = 3, P = 0.02), but the levels of EB3 protein were not affected significantly (Fig. 6A,B).

Lentiviral EB3 overexpression in WT neurons leads to increase in mushroom dendritic spines fraction (Fig. 4C). Is EB3 overexpression able to restore mushroom dendritic spines deficiency observed in PS1-M146V-KI neurons? In order to determine it, we co-transfected PS1-M146V-KI and WT neurons with tdTomato and mouse EB3 expression construct at DIV7. At DIV17 cultures were fixed and analysis of dendritic spines morphology was performed by confocal microscopy (Fig. 6C). Analysis of spine shapes revealed that expression of EB3 in WT neurons resulted in an increase in mushroom spines fraction to 47.8 ± 2.4% (n = 20, *P* = 0.009) (Fig. 6E), which is in agreement with lenti-EB3 expression data (Fig. 4D). Expression of EB3 in PS1-M146V-KI neurons increased mushroom spines fraction to 47.1 ± 1.9% (n = 21, P = 0.00005) (Fig. 6E). These data are consistent with the ability of EB3 overexpression to rescue mushroom spine deficiency observed following STIM2 knockdown (Fig. 4D). In both WT and PS1-M146V-KI cultures EB3 overexpression does not affect average dendritic protrusions density (Fig. 6D).

## Discussion

### EB3 protein affects morphology of hippocampal dendritic spines

Recent research indicates that dynamic microtubules with EB3-capped plus-end enter dendritic spines, and the number and duration of these invasions is facilitated by neuronal activity^37,41–44^. Microtubule insertions are transient and last for several minutes, and in only approximately 1–2% of mushroom spines MTs were detected at the steady-state^37,41,42^. We demonstrated that EB3 is present in hippocampal synaptosomes (Fig. 1B), which is in agreement with previous findings showed the presence of this protein in postsynaptic density fraction^37^.

Entry of microtubules facilitates spine enlargement^43^. These insertions are triggered by influx of Ca^2+^ and F-actin polymerization in the head and base of the spines^44^. In order to investigate the importance of EB3 for spines morphology, we modulated EB3 expression levels in hippocampal neurons by overexpression and shRNA-mediated knockdown. EB3 silencing by shRNA induced appearance of filopodia-like structures and dramatically reduced fraction of stubby and mushroom spines (Fig. 4B,D). EB3 overexpression had an opposite effect and shifted dendritic spines type balance toward mature mushroom and stubby spines (Fig. 4B,D). Previous studies have shown that EB3 silencing resulted in reduction of dendritic spines density, without changes in spine shape^41^. In another study, it was demonstrated that knockdown of EB3 reduced the density of mushroom-shaped spines while overexpression of EB3 resulted in robust increase in their number^37^. Our results are in consistence with the previous findings by Jaworski at al. and suggest that EB3 protein plays an important role in formation and/or maintenance of mushroom dendritic spines.

### Role of STIM2-EB3 interaction in modulation of dendritic spines morphology

In the present study, we have shown that STIM2 interacts with EB3 via SxIP aminoacid motif in ER calcium dependent manner (Fig. 2C,D), and this protein association affects spine morphology. Disruption of this interaction by IP/NN mutation in STIM2 leads to significant reduction in the fraction of mushroom spines (Fig. 3A,C). EB3 overexpression rescued mushroom spines deficiency observed after STIM2 knockdown (Fig. 4B,D), while in contrast, STIM2 overexpression failed to rescue loss of mushroom spines observed in EB3-deficient neurons (Fig. 4B,D). Notably, STIM2 overexpression or pharmacological activation of hippocampal nSOC channel TRPC6^50^ does not result in increased spine nSOC or fraction of mushroom spines in wild type neurons. Therefore, nSOC components in wild type spines are already maximally engaged with endogenous STIM2. We further established spine nSOC is slightly, but significantly affected by manipulating EB3 expression levels (Fig. 5).

EB3 protein caps plus-end of dynamic microtubules and serves for recruitment of various +TIPs proteins to the growing microtubules ends^45^. Considering that MTs entries are transient^42^ and lead to spines enlargement^52^, it was proposed that they serve for special cargo transportation during synaptic plasticity^53^. What important cargos are carried by dynamic microtubules into spine? Actin-associated protein p140Cap is one such cargo^37^. Our data suggests that STIM2 protein may be another. It has been shown that interaction of EB1 and STIM1 controls ER movement through tip-attachment complex, when ER tubule elongates together with growing microtubule end^46^, and also regulates store-operated calcium entry in non-excitable cells^54^. We suggest that association of the homologues to mentioned above proteins STIM2 and EB3 in neurons will serve the same function. Studies of ER movement in spines of Purkinje neurons revealed that it occurs predominantly with help of Myosin-Va motor protein moving through actin cytoskeleton, but rare microtubule-dependent ER insertion into spines also occurred^55^. Interestingly, this rare ER microtubule-based insertions were always accompanied by dynamic microtubule entry and were completely abolished by treatment of microtubule destabilizing drug nocodazole in low-doses^55^. We propose that rare ER microtubule-based insertions in hippocampal spines are mediated by STIM2-EB3 interaction.

AMPA receptor-containing endosome trafficking in hippocampal spines was reported to depend on both microtubule and actin cytoskeleton, with actin cytoskeleton playing a major role^56^. It was suggested that MT-based entry of endosomes into spines was limited only by the MT growth speed, and occurs faster than actin-based entry^56^. Supporting this idea, inhibition of MTs dynamics by nocodazole resulted in a marked shift of the endosome velocity distribution profiles toward lower speeds^56^. Summarizing, we may propose that observed deleterious effects of EB3 silencing on spines are probably occur due to improper activity-dependent cargo delivery into spines. Detailed future investigations will be needed to determine the role of the proposed STIM2 and EB3-mediated ER entry in spine plasticity.

### Microtubules and synaptic loss in Alzheimer’s disease

Alzheimer disease is the disease of lost memories. We and others previously proposed that loss of mushroom spines may underlie cognitive decline during the progression of AD, and that restoration of normal dendritic spines morphology is a potential therapeutic approach for AD treatment^11–13^. Reduction of mushroom spines number has been demonstrated in several cellular and animal models of AD^14,15,17,57,58^ including PS1-M146V-KI model. In the present study, we showed that EB3 overexpression rescues mushroom dendritic spine deficiency in PS1-M146V-KI neurons (Fig. 5C,E). Therefore, influencing dynamic MT and dendritic spine nSOC through overexpression of +TIP binding partner EB3 showed promising result in AD mouse model.

AD has been associated with altered microtubule dynamics^59^. It is widely known, that AD is characterized by deposition of the insoluble tau protein aggregates, which one in normal state provides MT stabilization and modulates axonal transport^60^. This observation triggered investigation of possible beneficial effects of exogenous MT-stabilizing drugs administration during AD^61^. To date several brain-penetrating MT-stabilizing compounds including epothilone D^62^, CNDR-51657^62^ and dictyostatin^63^ were reported to show neuroprotective effects in AD and tauopathies mouse models. Application of epothilone D at a subnanomolar concentration was reported to reverse Aβ-induced dendritic spines loss^58^, providing another link between microtubules dynamics and spine changes in AD. MT-stabilizing drugs were extensively used in anti-cancer therapy and were reported to have a number of adverse side effects, which restrict their administration for AD treatment^61^. Nevertheless, emerging evidence of link between MT and dendritic spines indicate that development of MT-targeting drugs is potential strategy for treatment of AD and other neurodegenerative diseases. Targeting of neuron-specific MT-based signaling pathways may help to overcome toxicity of such compounds.

## Methods

### Animals

PS1-M146V knock-in mice were kindly provided by Hui Zheng (Baylor University, USA). The breeding colony of PS1-M146V-KI and wild type mice of the same strain (C57BL/6 J background, #000664) obtained from the Jackson Laboratory were established and maintained in a vivarium four-five per cage with a 12 hours light/dark cycle in the animal facility located in the Laboratory of Molecular Neurodegeneration in Peter the Great St. Petersburg Polytechnic University. All procedures involving mice were approved by the Institutional Animal Care and Use Committee of the Research Institute of Influenza Ministry of healthcare of the Russian Federation, in accord with the Ministry of agriculture of the Russian Federation guidelines.

### Plasmids and viruses

pCSCMA:tdTomatoplasmid was a gift from Gerhart Ryffel (Addgene plasmid #30530)^64^. pCMV-GCaMP5G plasmid was a gift from Douglas Kim & Loren Looger (Addgene plasmid # 31788)^65^. Mouse EB3 cDNA was purchased from Open Biosystems and used to generate EB3 lentiviral expression construct with FLAG-tag added to 5′ end by PCR. GST-STIM2-CT (aa248-C terminal) was generated by PCR and cloned into PGEX-KG vector. YFP-STIM2 was kindly provided by Dr. Jen Liou (University of Texas Southwestern Medical Center, USA), lenti-HA-STIM2 construct was provided by Dr. Suya Sun (Ruijin Hospital Affiliated to Shanghai Jiao Tong University School of Medicine, China). YFP-STIM2-LA/SS (L377S, A380S), YFP-STIM2-IP/NN (I688N, P689N) and GST-STIM2-IP/NN (I688N, P689N) mutations were generated by Q5 mutagenesis Kit (NEB, #E0554S). After mutagenesis, plasmids were fully sequenced to confirm required mutations and verify the absence of additional one. Control short hairpin RNA interference (shRNAi) (#SHC002), mouse EB3–shRNAi (#SHCLNG-NM_133350, #TRCN0000315588) and mouse STIM2–shRNAi (#SHCLNG-XM_132038, #TRCN0000204753) lentivirus shuttle constructs were obtained from Sigma.

### Primary hippocampal neuronal cultures

Wild type (WT) and PS1-M146V-KI primary cultures of dissociated hippocampal cells were prepared and maintained as previously described^66^. Briefly, hippocampus of postnatal day 0–1 mouse pups were digested with papain solution (30 min at 37 °C; Worthington, #3176), then dissociated with 5 mg/ml Deoxyribonuclease I (Sigma, #DN-25) solution. Neurons were plated in 24-well culture plate on 12 mm glass coverslips precoated with 1% poly-D-lysine (Sigma, #p-7886) in Neurobasal-A (Gibco, #10888022) medium supplemented with 2% B27 (Gibco, #17504044), 1% heat inactivated fetal bovine serum (FBS, Gibco, #10500064), 0.5 mM L-Glutamine (Gibco, #25030024) and maintained at 37 °C in a 5% CO_2_ incubator. At 7 and 14 day *in vitro* (DIV7) half of the medium was replaced with culture medium without FBS. At DIV17 WT and PS1-M146V-KI hippocampal primary neurons were fixed in 4% formaldehyde and 4% sucrose in PBS, pH 7.3, solution for further analysis.

### Calcium phosphate transfection of primary hippocampal cultures

For assessment of synapse morphology, WT and PS1-M146V-KI hippocampal primary cultures were transfected with tdTomato plasmid or co-transected with tdTomato and STIM2, STIM2-LA/SS, STIM2-IP/NN, EB3 plasmid at DIV7 using the calcium phosphate method. Calcium transfection kit was purchased from Clontech (#631312). For transfection, conditioned medium was removed from cultures, and neurons were incubated in serum free medium with the calcium-phosphate-DNA precipitates formed in HEPES-buffered saline for approximately 1 hour. After completion of the transfection incubation period DNA-Ca2+ -phosphate precipitates were dissolved by incubating the cells in slightly acidic medium (10% CO2) without serum for a brief period of time. Cells were then returned into conditioned medium in 5% CO2 incubator and analyzed 9 days after transfection. Estimated efficiency of calcium phosphate transfection was ~1–5%.

### Dendritic spine analysis in primary hippocampal neuronal cultures

A Z-stack of 6–8 optical sections with 0.2 μm interval was captured using 100x oil objective (1.4 NA Olympus, UPlanSApo) with a confocal microscope (ThorLabs, USA). Each image was captured at 1024 × 1024 pixels with maximal resolution 0.107 μm/pixel and averaged nine times in real time. At least 18 cultured neurons from 3 batches of cultures were used for quantitative analysis in each experimental group. Dendritic protrusions in cultured primary hippocampal neurons were classified as mushroom, stubby, thin spines and filopodia. Quantitative analysis of dendritic spines was performed by using freely available NeuroStudio software package^67^ as described previously^14^. In classification of spine shapes we used the following cutoff values: aspect ratio for thin spines (AR thin) = 2.5, head to neck ratio (HNR) = 1.4, and head diameter (HD (crit)) = 0.5 μm. These values were defined and calculated exactly as described in^67^.

### Immunochemistry

DIV17 WT hippocampal primary neurons were fixed in 4% formaldehyde and 4% sucrose in PBS, pH 7.3, solution for 20 minutes at 4 °C, washed three times with PBS and permeabilized in 0.1% Triton X-100 in PBS solution for 5 minutes at room temperature. Nonspecific binding was blocked by incubating cells in 5% bovine serum albumin (BSA) in PBS solution for 1 hour. Then cells were incubated with anti-MAP2 (1:1000, Millipore, #MAB378), anti-EB2 (1:500, Sigma, #WH0010982M3) and anti-EB3 (1:500, Sigma, #SAB4200606) primary antibodies diluted in 2.5% BSA in PBS at 4 °C overnight. After three times wash in PBS, cells were incubated in 2.5% BSA in PBS solution with the secondary antibody (1:1000, Alexa Fluor 488, #A11008 or 564,#A11005, Invitrogen) for 1 hour at room temperature and then washed three times in PBS and visualized by a confocal microscope (ThorLabs).

### Lentivirus generation and infection of primary neuronal cultures

HEK293T line cells with 50–70% of confluency were co-transfected with shuttle (lenti-FLAG-EB3, lenti-HA-STIM2, lenti-shRNAi) and two helper plasmids pCMVΔ8.9 and pVSVg using polyethylenimine reagent (Polyscience, # 23966). 48–72 hours after transfection culture medium was collected, centrifuged 5 minutes at 2000 rpm, filtered through 0.45 μm pore, immediately frozen in liquid nitrogen and then stored at −80 °C. Each batch of generated lentiviruses was tested by Western blot in hippocampal neuronal culture infection experiments and the titer with minimal toxicity and maximum infection efficiency was used in all experiments. Lentivirus containing medium volume of 80–200 μl was added per well to WT hippocampal neuronal cultures at DIV8 (next day after calcium phosphate transfection).

### Crude synaptosome fraction

Whole brain or hippocampal regions were extracted from 3-month-old WT mice, homogenized in 0.32 M sucrose and 25 mM HEPES buffer, pH 7.2, on ice and centrifuged for 10 minutes at 800 g to remove the nuclei (P1). Supernatant from previous step (S1) was then centrifuged for 20 minutes at 12,000 g to separate synaptosomal supernatant (S2) and synaptosomal membrane fractions (P2). P2 pellet was solubilized in lysis buffer suitable for further applications for 2 hours at 4 °C. Insoluble material was removed by centrifugation of samples for 20 minutes at 16,300 g. All centrifugations steps were performed at 4 °C. Prepared synaptosomal fraction lysates were validated by Western blot analysis of expression of synaptic markers as CaMKII and pCaMKII (Fig. S2).

### Western blot analyses

For Western blot analysis crude synaptosome fraction or primary hippocampal neurons on DIV17 were homogenized in RIPA lysis buffer (50 mM Tris-HCl, 150 mM NaCl, pH 7.5, 0.1% sodium dodecyl sulfate, 0.5% sodium deoxycholate, 1% NP-40, 1 mM PMSF, protease (Sigma, #S8820) and phosphatase (Sigma, #P0044) inhibitors. The total protein lysates were separated by SDS-PAGE and analyzed by Western blot with anti-STIM2 (1:2000, AnaSpec, #54681), anti-Phospho-CaMKII (1:1000, Abcam, #ab171095), anti-CaMKII (1:2000, Chemicon, MAB8699), anti-EB2 (1:2000, Sigma, ##WH0010982M3), anti-EB3 (1:2000, Sigma, #SAB4200606), anti-actin (1:2000, Millipore, #MAB1501) and HRP-conjugated anti-rabbit (1:2000, #P0448) and anti-mouse (1:2000, #P0447) secondary antibodies from DAKO. Quantitative analysis of protein expression was performed using Quantity One software from BioRad. The mean density of each band was normalized to actin signal in the same sample and averaged. All Western blots were replicated 3–5 times.

### GST pull-down assay

GST-fusion proteins were expressed in BL21 E.coli strain and purified as described previously^68^. Expression level and purity of these proteins was evaluated based on coomassie brilliant blue-stained gel (Fig. S3). Crude synaptosome whole brain fraction was lysed in buffer containing 1% Triton, 130 mM NaCl, 50 mM Tris, pH 7.2, protease (Sigma, #S8820) and phosphatase (Sigma, #P0044) inhibitors. Protein extracts were incubated overnight at 4 °C with the corresponding GST-fusion proteins immobilized on glutathion-agarose beads (Sigma, #G4510). Then beads were washed four times with the 0.1% Triton X-100, 130 mM NaCl, 50 mM Tris, pH 7.2, buffer and boiled in sample buffer for 7 minutes at 95 °C. After boiling supernatant was collected, separated by SDS-PAGE and probed with the anti-EB3 antibody (1:2000, Sigma, #SAB4200606).

### Co-immunoprecipitation

For co-immunoprecipitation P2 pellet or HEK293T cells was solubilized in RIPA lysis buffer. For each co-immunoprecipitation reaction 25 μl Protein A/G agarose beads (Santa-Cruze, #sc-2003) were incubated with 2 μg primary anti-EB3 antibody (1:2000, Sigma, #SAB4200606) or normal rabbit IgG serum (Santa-Cruz, #sc-2027) for 1 hour at 4 °C, then with 350 µl of total protein lysate overnight at 4 °C on a rocking platform. Precipitated samples were washed three times with RIPA lysis buffer and final beads pellet was boiled in sample buffer for 7 minutes at 95 °C. After boiling supernatant was collected, separated by SDS-PAGE and probed with the anti-STIM2 antibody (1:2000, Anaspec, #54681).

### GCamp5.3 Ca^2+^ imaging experiments

Cultured WT hippocampal neurons were transfected with GCaMP5.3 expression plasmid or co-transfected with GCaMP5.3 and shControl, shEB3, FLAG-EB3 expression plasmids in 1:3 ratio at DIV7 using calcium phosphate method described earlier. At DIV15 GCaMP5.3 live fluorescent images were collected every 2 seconds using ThorLabs confocal microscope with 60x objective (1 NA Olympus, LUMPlanFNL) under control of ThorImgLS1.5 software. Neurons were incubated in Ca^2+^-free aCSF (Artificial celebrospinal fluid: 140 mМ NaCl, 5mМ KCl, 1 mМ MgCl2, 10 mМ HEPES, pH = 7.3) with 0.4 mM EGTA, 1 µM Thapsigargin and Ca^2+^ channels inhibitor mixture (1 µM TTX, 50 µM AP-5, 10 µM CNQX and 50 µM nifedipine) for 30 minutes. After recording basal fluorescent signals in Ca^2+^-free aCSF for 30 seconds nSOC was induced by puff application of 5 µl 2 M Ca^2+^ solution. Analysis of the data was performed using ImageJ software. The ROI used in the image analysis was chosen to correspond to spines, and signal was normalized to baseline.

### Statistical analyses

The results are presented as mean ± SEM. Statistical comparisons of results obtained in experiments were performed by Student’s t-test for two-group comparisons and one-way ANOVA for multiple comparisons between more than two groups. The *P* values are indicated in the text and Fig. legends as appropriate.

### Data availability

All data generated during this study are included in this published article (and its Supplementary Information files).

## Electronic supplementary material

Supplementary Figs 1 - 4
