# Supplementary material for: Stim2-Eb3 Association and Morphology of Dendritic Spines in Hippocampal Neurons
Source: Sci Rep. 2017 Dec 15;7:17625. doi: 10.1038/s41598-017-17762-8 (PMC5732248; doi:10.1038/s41598-017-17762-8)
Supplement: Supplementary file 1 — Supplementary Figs 1 - 4 [file 41598_2017_17762_MOESM1_ESM.pdf]

Supplementary Information

STIM2-EB3 association and morphology of dendritic spines in hippocampal neurons

Ekaterina Pchitskaya, Nina Kraskovskaya, Daria Chernyuk, Elena Popugaeva, Hua Zhang, Olga Vlasova, Ilya Bezprozvanny

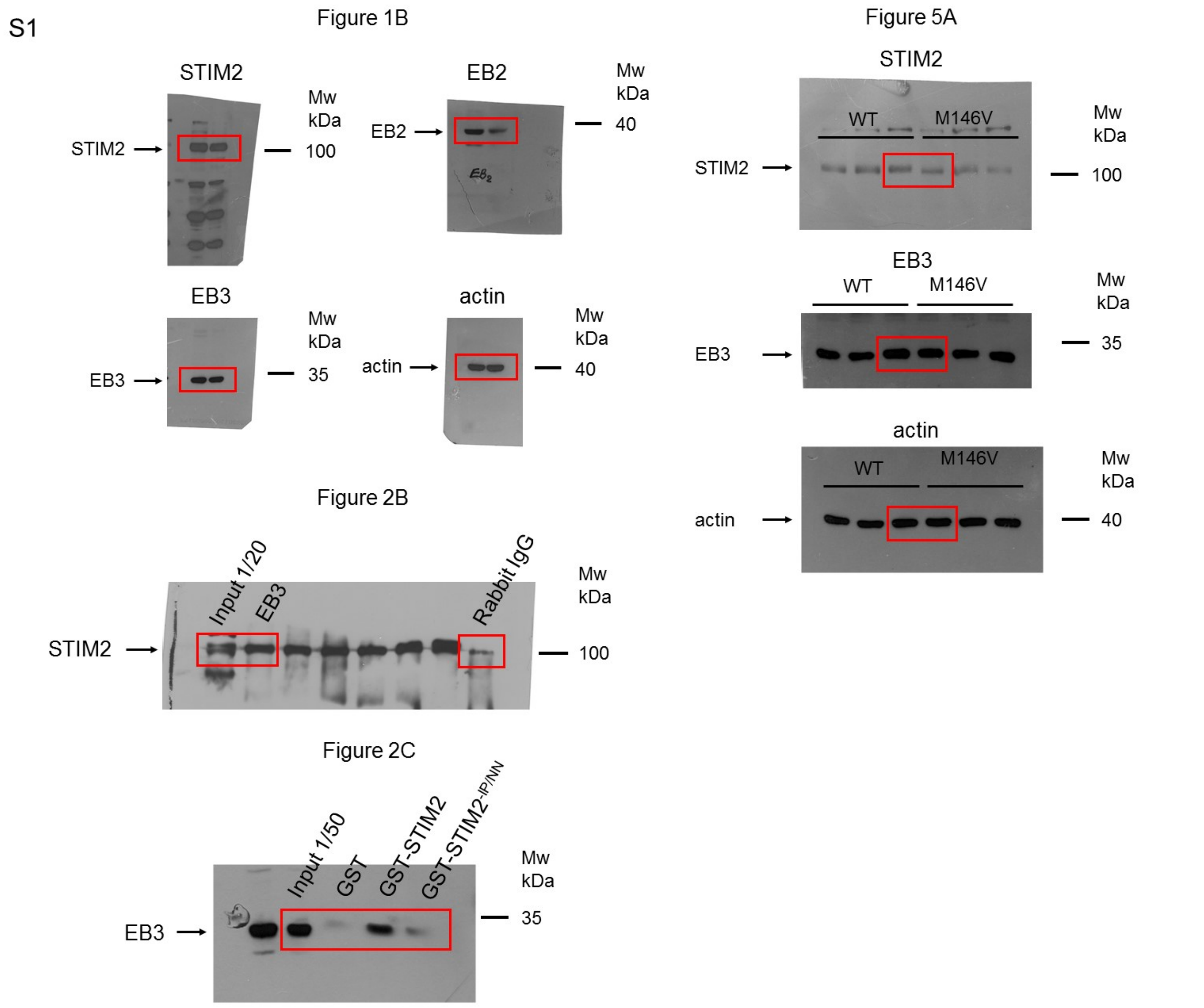

Fig. S1. Full-length blots corresponding to crop blots presented in main paper

Full-length blots for Figures 1B, 2B, 2C, 5A. Area displayed in main paper delineated by red rectangle.

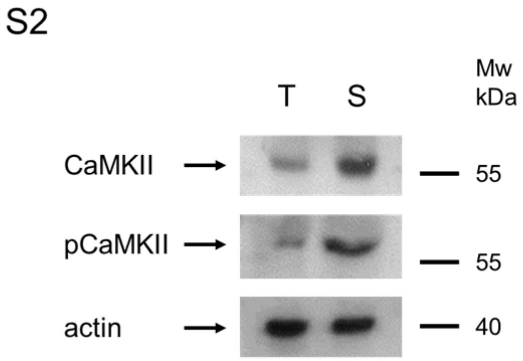

Fig. S2. Validation of crude synaptosomal fraction lysates preparation

Western blot analysis of synaptic marker proteins CaMKII and pCaMKII expression levels in total brain lysate (T) and crude whole brain synaptosomal fraction lysates (S). Related to Fig 1B.

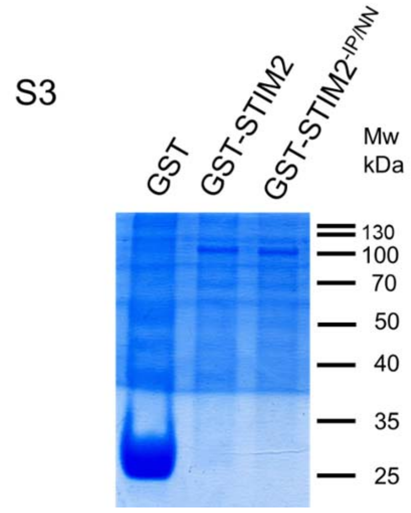

Fig. S3. Coomassie brilliant blue-stained gel loaded with proteins used for GST pull-down experiments

Coomassie-stained gels are shown for purified proteins GST and GST-STIM2, GST-STIM2-IP/NN fusions used for GST pull-down experiments. Related to Fig 2C.

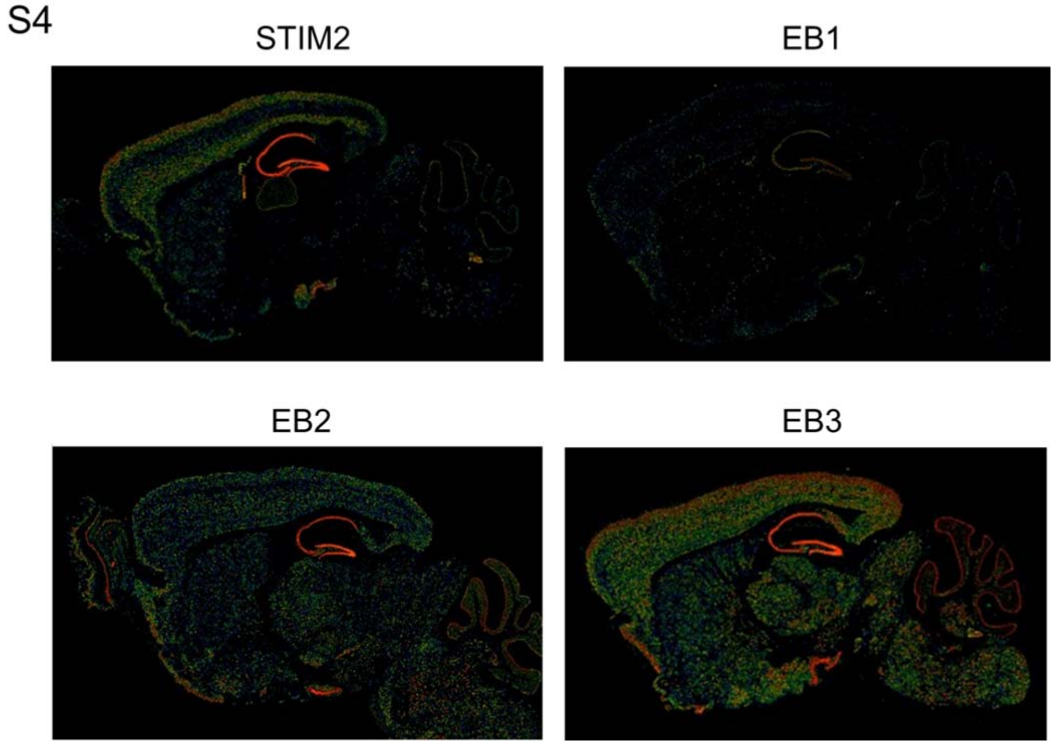

Fig. S4. STIM2 and EB proteins expression in mouse brain (Related to Fig. 1).

In situ hybridization images from Allen Brain Atlas demonstrating expression of STIM2 and EB proteins in mouse brain.
